# Dynamic adaptive X-ray optics. Part II. High-speed piezoelectric bimorph deformable Kirkpatrick–Baez mirrors for rapid variation of the 2D size and shape of X-ray beams

**DOI:** 10.1107/S1600577518015965

**Published:** 2019-01-01

**Authors:** Simon G. Alcock, Ioana-Theodora Nistea, Riccardo Signorato, Robin L. Owen, Daniel Axford, John P. Sutter, Andrew Foster, Kawal Sawhney

**Affiliations:** a Diamond Light Source, Harwell Science and Innovation Campus, Didcot, Oxfordshire OX11 0DE, UK; b S.RI. Tech s.r.l.s, Viale del Lavoro 42A, 35010 Vigonza, Padova, Italy

**Keywords:** adaptive X-ray optics, deformable piezoelectric bimorph mirrors, high-voltage bipolar power supplies, Kirkpatrick–Baez mirrors, Fizeau interferometry

## Abstract

The tangential curvature of actively bent X-ray mirrors at synchrotron radiation and X-ray free-electron laser (XFEL) facilities is typically only changed every few hours or even days. This operation can take tens of minutes for active optics with multiple bending actuators and often requires expert guidance using in situ monitoring devices. Hence, the dynamic performance of active X-ray optics for synchrotron beamlines has historically not been exploited. This is in stark contrast to many other scientific fields. However, many areas of synchrotron radiation and XFEL science, including macromolecular crystallography, could greatly benefit from the ability to change the size and shape of the X-ray beam rapidly and continuously. The advantages of this innovative approach are twofold: a large reduction in the dead time required to change the size of the X-ray beam for different-sized samples and the possibility of making multiple changes to the beam during the measurement of a single sample. In the preceding paper [Part I; Alcock, Nistea, Signorato & Sawhney (2019 ▸), *J. Synchrotron Rad.* **26**, 36–44], which accompanies this article, high-speed visible-light Fizeau interferometry was used to identify the factors which influence the dynamic bending behaviour of piezoelectric bimorph deformable X-ray mirrors. Building upon this ex situ metrology study, provided here is the first synchrotron radiation beamline implementation of high-speed adaptive X-ray optics using two bimorphs operating as a Kirkpatrick–Baez pair. With optimized substrates, novel opto-mechanical holders and a next-generation high-voltage power supply, the size of an X-ray beam was rapidly and repeatedly switched in <10 s. Of equal importance, it is also shown that compensation of piezoelectric creep ensures that the X-ray beam size remains stable for more than 1 h after making a major change. The era of high-speed adaptive X-ray optics for synchrotron radiation and XFEL beamlines has begun.

## Abstract 2

Demonstrated here is the first simultaneous high-speed beamline operation of a pair of piezoelectric bimorph deformable Kirkpatrick–Baez mirrors for the rapid and repeatable change and stabilization of the vertical and horizontal size of a synchrotron X-ray beam within only a few seconds. This enables continuous adaptive shaping of the X-ray beam in almost real time. Such innovations could lead to a major scientific change in how deformable X-ray mirrors are used at synchrotron and X-ray free-electron laser sources.

## 1. Introduction   

Active and adaptive optics have routinely been used for several decades by many scientific disciplines, including astronomy, laser physics and microscopy. Unlike passive optics with fixed pre-polished surface profiles, active and adaptive optics can be deformed to suit a variety of experimental configurations. In this paper, we follow the convention that active optics are quasi-statically bent to a given shape, whereas adaptive optics are continuously tuned, often at high speed, based on closed-loop feedback. For active optics, multiple force actuators, used collectively, bend the optical surface of piezoelectric or mechanical deformable mirrors to a range of shapes and curvatures. Individual actuators can also counter­act localized optical distortions which are permanent or slowly varying in time. This includes polishing errors, mounting strains and thermal drifts. With adaptive optics, such actuators operate more quickly and can correct fast disturbances in the incoming photon-beam wavefront. Alternatively, they can rapidly redirect or modify the shape of the reflected photon beam. For example, adaptive optics for ground-based astronomy are driven in closed loop at high speed using real-time feedback to correct wavefront aberrations caused by atmospheric turbulence.

Grazing-incidence X-ray mirrors at synchrotron radiation and X-ray free-electron laser (XFEL) facilities operate in vacuo and are generally not subjected to fast-changing external influences. After mirror assembly and installation on a beamline, distortions of the optical surface caused by polishing errors, vacuum forces, gravitational sag and mechanical clamping are usually constant in time. Since many synchrotron facilities now operate with the electron beam at fixed current, via top-up mode, a constant photon heat load illuminates the X-ray optics. Therefore, there are generally negligible dynamic changes to the X-ray wavefront caused by heating or cooling of upstream optics. With no compelling necessity for the rapid correction of dynamic disturbances, synchrotron active optics have traditionally been used in a ‘set and forget’ mode: after initial optimization, modifications are made only every few hours or days to change the X-ray beam size or focal distance. In some instances, active mirrors are not adjusted for many weeks, or even months. Therefore, historically, adaptive X-ray optics have generally not been utilized at synchrotron radiation and XFEL facilities.

However, in recent years, there has been a speed revolution driven by increasing scientific demands from the user community. With ever brighter X-ray sources, faster detectors, robot-controlled sample changers and automated data-acquisition and analysis software, samples can now be loaded, aligned and scanned at unprecedented speeds. Many beamlines, especially those used for macromolecular crystallography (MX), are now capable of routinely characterizing hundreds of samples per day (Aller, 2015 ▸; Grimes *et al.*, 2018 ▸). As well as technical merit, such beamlines are also judged by their scanning efficiency and data output, so every second counts. To exploit the high brilliance of modern X-ray sources efficiently, the size of the beam should be matched to that of the sample. Alternatively, variable-sized X-ray beams can probe sub-domains of larger samples. In both cases, sample-to-sample variation means that a different X-ray beam size may be required for each data-collection run. Quick changes to the size of the X-ray beam are routinely made by varying the width of beam-defining apertures. However, this detrimentally reduces the photon flux. Changes in X-ray beam size can also be achieved by modifying the surface profile of achromatic reflective optics or adding chromatic refractive lenses. These approaches have the advantage of preserving the total flux at the sample position. But manipulating X-ray optics using mechanical actuators also has several disadvantages: it is typically much slower than using apertures, expert intervention may be required, opto-mechanical holders and control systems are not optimized for fast operation, and there can be motor heating and wear issues for fast and repeated use. These factors motivate the search for novel optics and control systems which can rapidly manipulate the size and shape of the full X-ray beam without suffering from the aforementioned drawbacks.

Piezoelectric bimorph deformable X-ray mirrors (Susini *et al.*, 1996 ▸; Signorato *et al.*, 1998 ▸; Alcock *et al.*, 2013 ▸, 2015 ▸; Vannoni *et al.*, 2014 ▸; Ning *et al.*, 2007 ▸), so-called bimorphs, are a promising candidate for high-speed and repeatable operation. Piezoelectrics are known to respond rapidly and accurately to voltage impulses. They also do not dissipate a significant heat load, even when used with a high duty cycle in vacuum and without cooling. Many scientific disciplines routinely operate bimorph mirrors at refresh rates >100 Hz, albeit not in vacuum and with a significantly relaxed tolerance on the amplitude of surface errors (due to the proportionate wavelength of visible light and air turbulence cells) (Garcia, 1982 ▸; Madec, 2012 ▸). The intrinsic repeatability of piezoelectric actuators also makes it possible to drive bimorph mirrors in open loop, using pre-determined look-up tables of voltages (Huang, 2011 ▸), without the necessity of using sophisticated metrology feedback systems.

The ultimate challenge for adaptive X-ray optics in the synchrotron radiation and XFEL domain is achieving a fast refresh rate, in combination with nanoscale spatial resolution and stability. Conventional MX data collection times at synchrotron radiation beamlines are of the order of 1–60 s, serial collections take 10–100 ms and conventional sample exchange takes ∼20 s. Therefore, an initial refresh rate of 0.1 to 1 Hz could sensibly provide a suitable variation in the size of the X-ray beam to match the typical frequency and duration of sample change and data collection.

X-ray bimorphs for synchrotron applications are known to take >15 min to stabilize on the nanometre scale and their curvature may drift by a few percent for several hours afterwards (Alcock *et al.*, 2015 ▸). Stick/slip behaviour of the substrate mounted in a non-ideal holder can also cause the curvature of the mirror to change when a voltage is applied to individual piezo electrodes or collectively to all (Vannoni *et al.*, 2016 ▸). Such waiting times are acceptable when beamlines operate with quasi-static optics, tuned to a known set point and then left untouched for prolonged periods. The final status of the mirror is then extremely stable and is not influenced by external parameters such as temperature. In such cases, the only criterion of merit is repeatably achieving a specific focus using a given set of input voltages stored in a look-up table. There are anecdotal examples of beamlines operating with the same set of voltages applied to their bimorphs for several years, without noticing significant degradation of the focused X-ray beam. The only precautions after an X-ray source shutdown are to align the mirror and apply voltages at least 1 h before providing X-ray beam to the scientific users. But what if users demand quick changes to the X-ray beam size or focal position? Clearly, this is not easily achieved with older bimorphs. However, these shortcomings are not an intrinsic limitation of bimorph technology.

**Figure 1:**
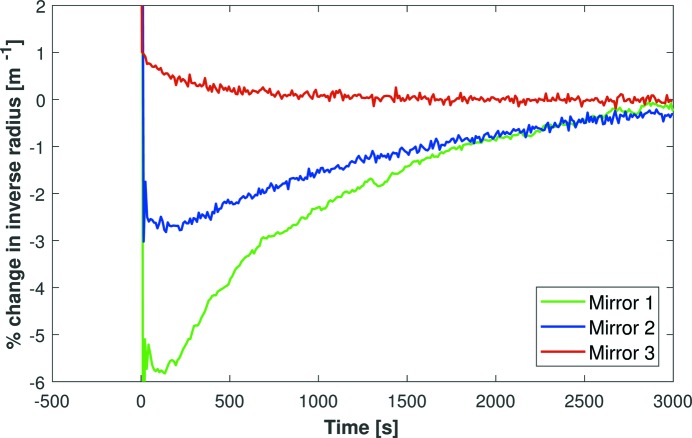
Fizeau interferometer measurement of the inverse radius of curvature of three micro-focus bimorph mirrors as a function of time after a 1000 V impulse was applied to the piezoelectric actuators. Mirrors 1 and 2 are old-fashioned bimorph mirrors which drift in curvature over time. Mirror 3, a new optic mounted in a strain-free holder, exhibits significantly less drift and has subsequently been installed on the I24 beamline at Diamond as a horizontally focusing mirror. Curvature data are plotted as a percentage change relative to each mirror’s steady state. Stabilization in open loop to within <1% is achieved in 20 s for Mirror 3. This proves that optimized bimorph deformable mirrors are intrinsically capable of being rapidly changed and stabilized in only a few seconds.

As described in the preceding paper (Part I; Alcock *et al.*, 2019 ▸), high-speed Fizeau interferometry was used to identify the main factors which influence the bending behaviour of micro-focusing bimorph X-ray mirrors. After applying a major impulse (1000 V) to all piezos, dynamic changes in the inverse radius of curvature were measured for three mirrors. Fig. 1 ▸ shows the percentage change relative to the steady-state curvature of each mirror (time *t* → ∞). Mirror 1 (green curve) is an obsolete first-generation bimorph with rigid electrical connectors and an over-constraining opto-mechanical holder. Mirror 2 (blue curve) is similar to Mirror 1, but has flexible wires rather than rigid electrical connectors to supply voltages to the piezo electrodes. Mirror 3 is a novel second-generation bimorph (red curve) with a custom-designed strain-free kinematic holder. The advantages of using flexible wires and an upgraded opto-mechanical holder are apparent, since Mirrors 1 and 2 take >1500 s to stabilize to within 1% of the final curvature, whereas Mirror 3 stabilizes to <1% within 20 s.

An important point about Fig. 1 ▸ is that the curvature drift for Mirror 3 is in the opposite direction to the other two mirrors and is as expected for intrinsic piezo creep. This indicates that Mirrors 1 and 2 are dominated by mechanical resistive forces from the rigid electrical connectors and/or the holders. Conversely, these factors have been successfully minimized for Mirror 3 and the small amount of residual drift can be solely attributed to piezoelectric creep. Therefore, using upgraded opto-mechanics and a programmable high-voltage power supply, we were able to make major changes to the curvature of a bimorph in <10 s, without subsequent drift. The positive outcome of this ex situ metrology study brought us naturally to the next step: install Mirror 3 on a synchrotron beamline at Diamond Light Source and confirm that the X-ray beam size can be rapidly changed and stabilized in a repeatable manner, thereby enabling scientific users to access this functionality during routine beamline operation.

## 2. Experimental   

### 2.1. Bimorph deformable mirrors   

Mirror 3 (a horizontally focusing mirror, HFM) and its vertically focusing (VFM) Kirkpatrick–Baez (KB) partner were procured for the Microfocus Macromolecular Crystallography beamline (I24) at Diamond Light Source. Two fused silica substrates were super-polished using elastic emission machining (EEM) at JTEC, Japan, and converted into second-generation bimorph X-ray mirrors (Alcock *et al.*, 2015 ▸) by glueing piezoelectric ceramic bars to the side faces of the substrates at Thales-SESO (TSESO), France. The overall design and project coordination were managed in a collaboration between Diamond and S.RI. Tech, Italy. Both substrates are coated with a metallic bilayer (4 nm of rhodium deposited over 25 nm of platinum) for enhanced reflectivity over an extended X-ray energy range of 6 to 25 keV. Each mirror is 240 mm long and has 16 independent piezoelectric actuators. The VFM has the added complexity of operating facing downwards, which made the design and metrology testing more challenging. After piezo voltage corrections using ex situ metrology feedback from the Diamond-NOM (nanometre optical metrology) instrument (Alcock *et al.*, 2010 ▸), slope errors of ∼180 nrad r.m.s. were obtained for each fully assembled optic for a range of ellipses to suit the beamline optical geometry. The VFM was one of the first mirrors to be characterized facing downwards on the upgraded Diamond-NOM slope profiler in the Optical Metrology Laboratory at Diamond. Improved stress-free kinematic holders were designed by S.RI. Tech and manufactured by CINEL, Italy, to minimize the magnitude of mechanical drift previously observed with earlier bimorph mirrors. TSESO’s newest flexible wiring scheme was used to minimize strains applied to the mirrors.

### 2.2. High-voltage bipolar power supply   

As shown in Fig. 1 ▸, bimorphs can be rapidly stabilized to a new curvature if care is taken to optimize the mechanical strain. However, the time taken to apply the required voltage also needs to be considered for large changes in curvature. This period is dictated by the voltage slew rate (volts per second) applied by the power supply to the bimorph. To the best of the authors’ knowledge, most X-ray bimorphs are typically driven with slew rates of ∼10 V s^−1^. But, to create a dramatic change in the size of an X-ray beam, a voltage change of >1000 V may be necessary. Using historic slew rates, such a voltage ramp would take >2 min, which already exceeds our target time for stabilization. This issue has recently been addressed by the development and commercial availability of the HV-ADAPTOS, a new high-voltage bipolar power supply, manufactured by CAEN, Italy, and distributed by S.RI. Tech. Aside from a significantly higher voltage slew rate, the HV-ADAPTOS software now allows simultaneous operation of more than one bimorph. This enables the X-ray beam size to be simultaneously changed in the vertical and horizontal directions by synchronously supplying voltages to the HFM and VFM of a KB pair.

### 2.3. Characterization of X-ray performance   

X-ray measurements of the micro-focus KB bimorphs were performed on the I24 beamline using X-rays with an energy of 12.8 keV. A two-stage focusing arrangement with zoom capabilities, utilizing two pairs of KB mirrors (pre- and micro-focus), creates an X-ray beam of ∼6 µm × 7 µm (horizontal × vertical) FWHM at the sample position. This provides a flux of ∼3 × 10^12^ photons s^−1^. Changes in X-ray beam size were monitored by placing a 25 µm-thick yttrium aluminium garnet (YAG) scintillator (from Crytur) at the sample position. Images of the scintillator were recorded using an on-axis viewing CCD system with an exposure time of 50 ms, thereby removing possible beam-broadening effects from vibrations below 20 Hz. The X-ray beam was attenuated to 0.3% of the total flux to avoid saturating the CCD camera. 2D beam widths were automatically calculated and recorded in real time using the EPICS *AreaDetector* software operating at 10 frames s^−1^. This approach provides reliable information on dynamic changes in the X-ray beam profile. However, due to the unavoidable convolution of the non-negligible broadening effect of the YAG scintillator, this method does not provide an accurate measurement of the size of the X-ray beam. For an absolute measurement of beam size, knife-edge scans were performed at the sample position in a stepwise manner using a 250 µm-diameter gold wire mounted on motorized micro-positioning stages. Using this method, which takes ∼2 min per scan, the FWHMs of the vertically focused and defocused beams were measured to be 6.7 and 50 µm, respectively. This compares with FWHMs of 13 and 55 µm, respectively, recorded using the YAG scintillator and CCD camera.

## 3. Results   

### 3.1. X-ray beam-size monitoring   

To demonstrate the repeatability of the <7 µm focus, a large voltage increase of +500 V was uniformly added to all electrodes to produce a defocused beam of ∼50 µm FWHM. For these proof-of-principle experiments, no special care was taken to control the overall quality or shape of the defocused X-ray beam; we simply wanted to assess repeatability. Plans are underway to develop the HV-ADAPTOS software further to create a smooth defocused X-ray beam by applying unique optimized voltages to each piezo electrode, rather than the same voltage shift to all. This approach has previously created defocused X-ray beams with acceptable levels of striation using super-polished bimorphs (Sawhney *et al.*, 2010 ▸). The X-ray beam was then refocused by applying −500 V to all piezo electrodes to return them to their initial focusing values, whilst monitoring whether the beamline imaging systems could sufficiently resolve temporal changes in the size of the X-ray beam. If repeatability of focus is proven, we can then assume it will be straightforward to extend operation to an arbitrary number of focusing/defocusing configurations, pre-determined during commissioning experiments and stored as voltage look-up tables in the HV-ADAPTOS controller. Data shown in subsequent plots are recorded by making changes to the VFM, but results from the HFM were found to be comparable.

#### 3.1.1. Knife-edge scans   

**Figure 2:**
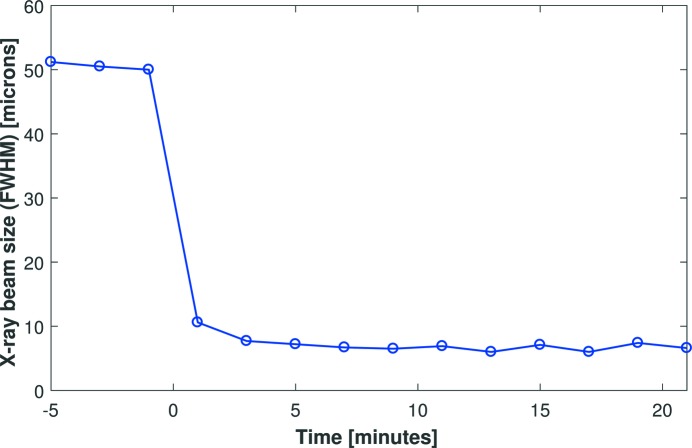
Dynamic measurement of the vertical size of the X-ray beam on the I24 beamline using knife-edge scans. This method provides an accurate measurement of the beam size but at the expense of reduced time sensitivity (the duration of each scan is ∼2 min). There is no discernible drift in the size of the X-ray beam, which illustrates the quality of the improved opto-mechanical holders for the bimorphs.

Fig. 2 ▸ shows the width of the X-ray beam as it was brought into focus, as measured using knife-edge scanning. Although piezo voltages were changed at *t* = 0, timings were not synchronized with the completion of knife-edge scans. This method accurately records the size of the X-ray beam but at the expense of strongly reduced temporal sensitivity (∼2 min to collect each scan). Knife-edge data show that the measured size of the X-ray beam is stabilized within the first few scans and remains so. This provides confidence that the opto-mechanical holder does not strongly influence the focusing power of the mirror. In reality, the measured size is an upper limit: beamline vibrations will smear out the X-ray beam during the acquisition period of each knife-edge scan. However, since each knife-edge scan takes ∼2 min to complete, this approach is unsuitable for accurately monitoring rapid changes in the X-ray beam size. For example, the X-ray beam was focused during the slit scan which completed at *t* ≃ 1 min in Fig. 2 ▸. The calculated beam size for this anomalous point is a weighted average of the defocused and focused beams and should be ignored. Finally, the knife-edge method can only scan in a single direction at a time, making it impossible to measure the 2D size of the X-ray beam simultaneously.

#### 3.1.2. Scintillator and CCD camera   

**Figure 3:**
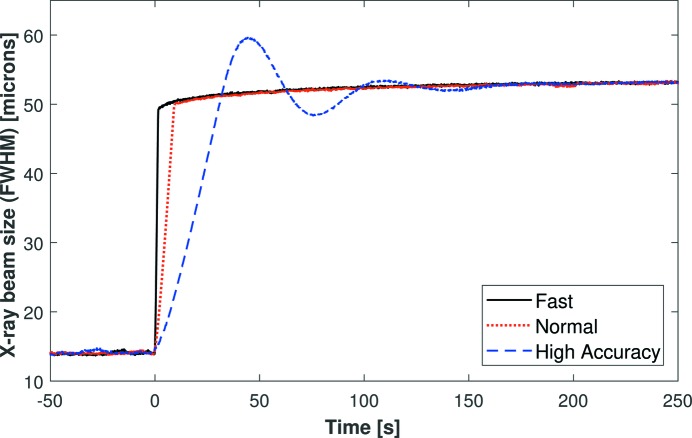
Dynamic measurement of the vertical size of the X-ray beam as a function of time as it was defocused by applying +500 V (at *t* = 0) to all piezos of I24’s vertical micro-focus bimorph mirror. Each curve shows the size of the X-ray beam with the high-voltage power supply operating in a different mode. Such results clearly indicate that the YAG scintillator/CCD camera can adequately resolve dynamic changes in the X-ray beam size. Such images of dynamic focusing are directly comparable with those captured by the Fizeau interferometer in Fig. 1 ▸.

Fig. 3 ▸ shows the variation in size of the X-ray beam as measured by the YAG scintillator/CCD camera as a function of time after applying +500 V to all electrodes to defocus the bimorph mirror. We chose to monitor the defocusing process since inherent broadening by the scintillator means that we are unable to characterize the size of the X-ray beam accurately below ∼10 µm. Three modes of operation were used for the HV power supply: normal (10 V s^−1^), fast (with an increased voltage slew rate of 300 V s^−1^) and high accuracy (where a damped sinusoidal voltage oscillation is deliberately applied immediately after the main voltage impulse). The damped voltage oscillations provided by the high-accuracy mode are clearly visible in Fig. 3 ▸, which confirms that the YAG scintillator/CCD camera can adequately resolve dynamic changes in the X-ray beam size. Additionally, the rate of drift in the size of the X-ray beam is not dependent on the voltage slew rate (normal = 10 V s^−1^ or fast = 300 V s^−1^). Such results are in excellent agreement with the earlier Fizeau interferometer study in Part I which monitored the dynamic curvature of each mirror. Therefore, thanks to their superior time resolution, the YAG-based data are shown in subsequent plots to demonstrate the relative change in the size of the X-ray beam.

### 3.2. Rapid changing of the 2D size of the X-ray beam   

**Figure 4:**
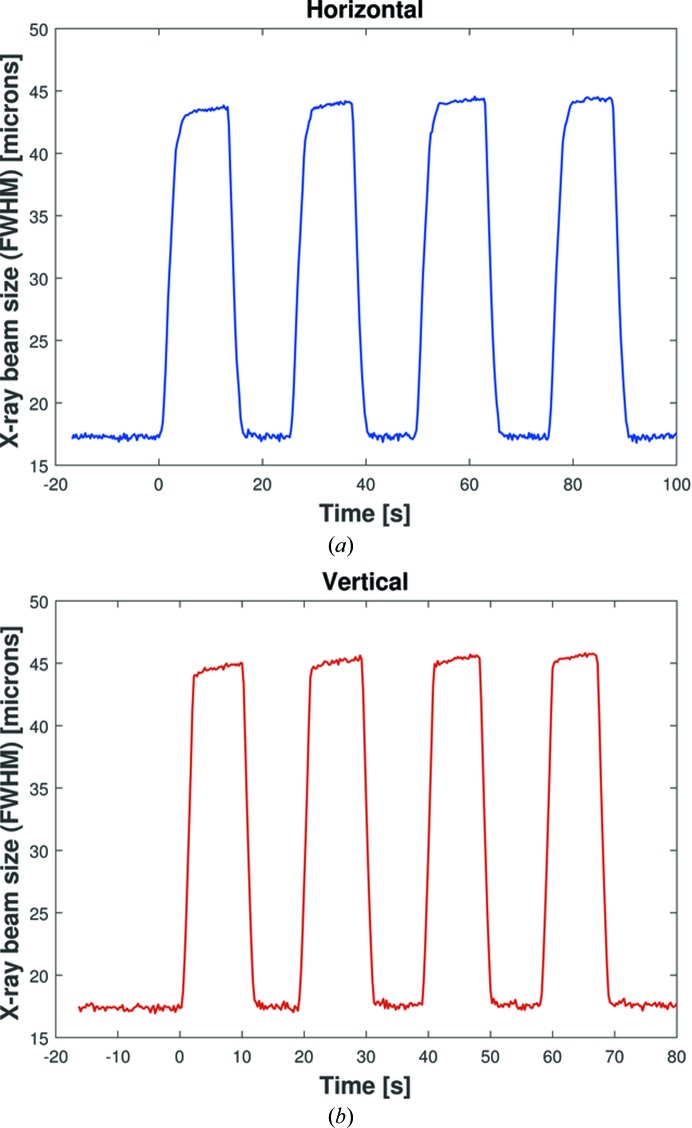
(*a*) Horizontal and (*b*) vertical sizes of the X-ray beam as a function of time, as measured by the I24 beamline’s scintillator and CCD camera. Voltage impulses of ±500 V were added to all piezo electrodes of each KB mirror to focus or defocus the X-ray beam in the horizontal or vertical direction simultaneously. The size of the beam is shown to be changeable in only a few seconds and is highly repeatable.

Using live snapshots of the YAG/CCD camera, the FWHM of the X-ray beam was automatically calculated in real time in the vertical and horizontal directions. Upgraded software for the HV-ADAPTOS now enables voltages to be applied simultaneously to both mirrors, which halves the time needed to make changes to a KB mirror pair. The parameters for voltage changes (such as slew rate and profile) are independent and freely selectable for each mirror. Hence, it is possible, simultaneously and independently, to focus and/or defocus in either or both directions. As a demonstration of these new features, Fig. 4 ▸ shows the 2D width of the X-ray beam during a series of rapid cycles of focusing and defocusing in the horizontal [Fig. 4 ▸(*a*)] and vertical [Fig. 4 ▸(*b*)] directions. This was achieved by simultaneously applying ±500 V to all piezo electrodes. This confirms that major changes can be repeatably made to the optical surface in just a few seconds.

### 3.3. Manually compensating for piezo creep   

**Figure 5:**
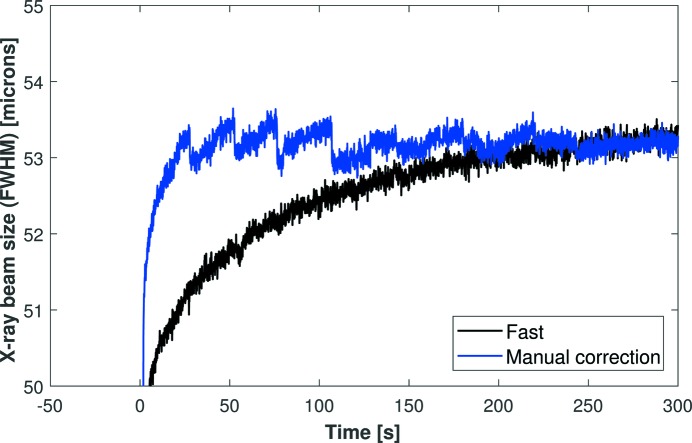
Dynamic measurement of the X-ray beam size with (blue curve) and without (black curve) manual compensation of voltages to reduce the effect of piezo creep. Jumps in the blue curve correspond to when small voltage corrections were applied to the mirror. With this simple correction scheme, the X-ray beam reaches 95% of its final size in less than 2 s, 98% within 3 s and 99% within 7 s. This clearly demonstrates that the size of the X-ray beam can be rapidly controlled and stabilized in just a few seconds.

The black curve in Fig. 5 ▸ shows how the size of the X-ray beam expands naturally due to piezoelectric creep after applying a uniform +500 V shift to all piezoelectric actuators to defocus the mirror. Using knowledge gained from the ex situ metrology study in Part I, we sought to counteract piezo creep, thereby minimizing the time taken to reach the final steady-state size of the X-ray beam. The compensation scheme consists of an initial pre-calculated overshoot beyond the target voltage, followed by a gradual user-defined reversion back towards the target voltage in small discrete steps. Using real-time feedback from the YAG scintillator/CCD camera, corrective voltage adjustments (steps of −2 V) were manually applied to all electrodes to compensate for piezoelectric creep and stabilize the size of the X-ray beam. The blue curve in Fig. 5 ▸ illustrates the dramatic improvement achieved using this simple creep-compensation method. Discrete jumps of a few hundred nanometres in the width of the X-ray beam are observed at the instant when small voltage corrections were applied to the mirror. Nevertheless, the data clearly show that the X-ray beam can be stabilized to within 1% of its final size in <7 s using manual creep compensation

### 3.4. Automatic compensation of piezo creep   

**Figure 6:**
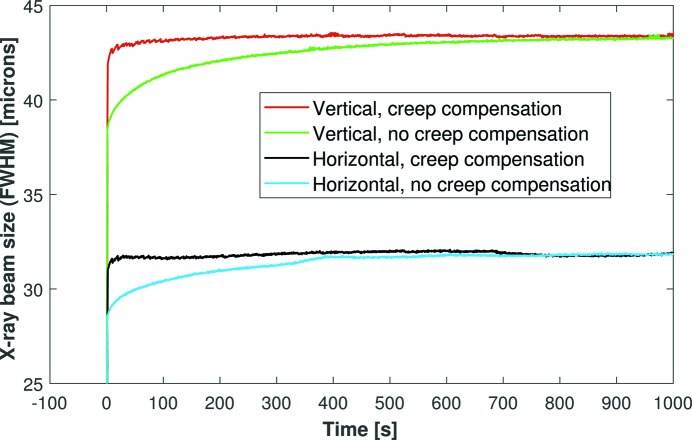
Simultaneous changes in the size of the X-ray beam in the horizontal and vertical directions, with and without automated piezo creep compensation, following synchronous defocusing of both mirrors by +500 V. Creep compensation provides a demonstrable improvement in the time required to stabilize the size of the X-ray beam. This is a dramatic improvement compared with the traditional usage of KB mirrors.

The minor jumps in the size of the X-ray beam can be further diminished by making more frequent, but smaller amplitude, voltage changes. To automate this process, the control firmware and graphical user interface of the HV-ADAPTOS power supply were modified by CAEN. This enables a list of correction voltages to be read in from a file and automatically applied at specified times (typically every few seconds) immediately after the main voltage ramp. Fig. 6 ▸ shows this automated scheme in action following a +500 V increment. With creep compensation, the X-ray beam stabilized in the horizontal direction to within 95% of the final size in <2 s, 97.5% in 2.5 s and 99% in 7.5 s. As a comparison, without creep compensation, horizontal stabilization occurred to 95, 97.5 and 99% within 73, 199 and 342 s, respectively. Similar values were found for the vertical direction. This confirms that the automated correction scheme virtually eliminates piezo creep and achieves almost instantaneous stabilization of the X-ray beam to the target size.

The great advantage of this correction scheme is that multiple X-ray measurements and the earlier ex situ metrology scans all confirm that piezo creep and the correction scheme are very repeatable for a given bimorph mirror in response to a specific voltage impulse. Therefore, unlike earlier work which utilized continuous in situ feedback (Poyneer *et al.*, 2014 ▸; Mimura, 2010 ▸), once a successful compensation recipe has been found, it can reliably be applied ‘blindly’ without the necessity for real-time monitoring of the X-ray beam or the mirror’s shape. In fact, compensation voltages can be pre-calculated by simply measuring the effects of piezo creep using ex situ metrology or X-ray monitoring during commissioning and using the fact that the bimorph’s radius of curvature is inversely proportional to the applied voltage.

**Figure 7:**
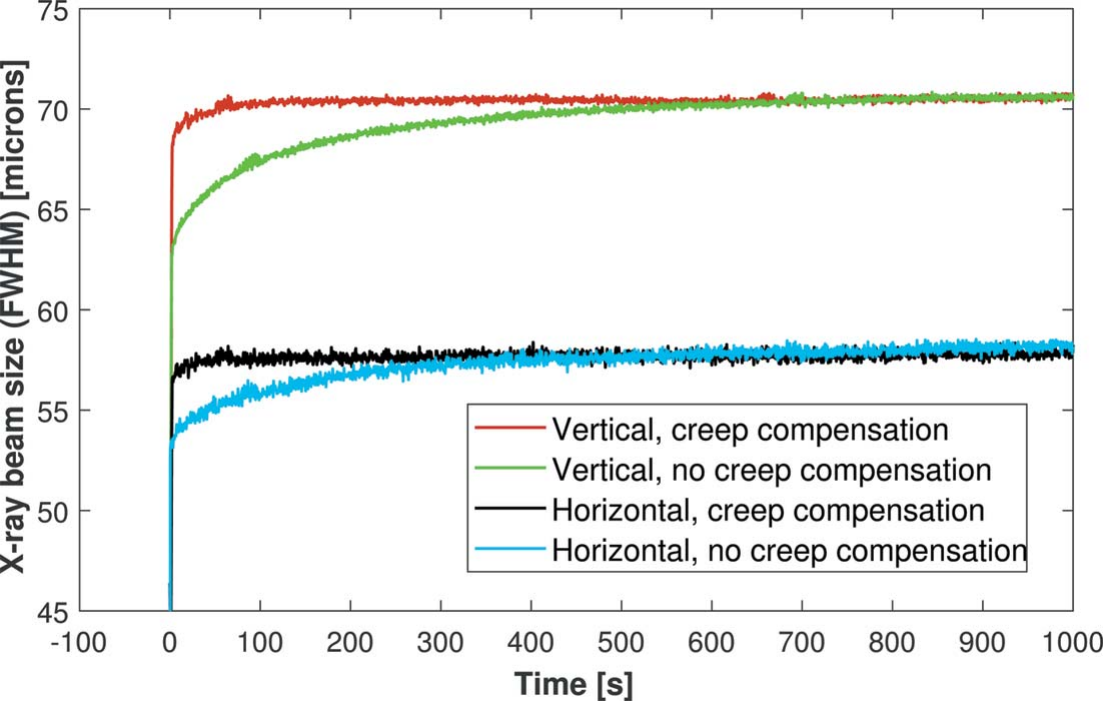
Repeat of the simultaneous defocusing X-ray tests shown in Fig. 6 ▸, but with a voltage jump of +800 V. The piezo creep-compensation voltages were simply 1.6 (800/500) times greater in amplitude than the +500 V tests. This confirms that the compensation voltages are proportional to the main voltage jump, which makes it very easy to pre-calculate the compensation voltages required for any permissible change in X-ray beam size.

It would be an extremely time-consuming exercise to determine experimentally the optimum correction voltages required to change the X-ray beam to any possible size. We therefore investigated whether the creep-correction voltages are linearly proportional to the main voltage jump. After refocusing the mirror, an +800 V step was simultaneously applied to all piezo electrodes, without creep compensation, whilst the 2D size of the X-ray beam was monitored (see Fig. 7 ▸). This process was then repeated, but with compensation voltages scaled to be 1.6 (*i.e.* the ratio of 800 V to 500 V) times greater in amplitude than the +500 V tests shown in Fig. 6 ▸. With creep compensation, the X-ray beam stabilized in the horizontal direction to within 95% of the final size in <2 s, 97.5% in 4 s and 99% in 18 s. These values could certainly be improved, but the excellent improvement in X-ray beam size confirms our simple hypothesis that the creep-correction voltages are linearly dependent on the amplitude of the voltage jump. This offers a quick and simple method of predicting the amount of creep compensation required for any given change in X-ray beam size. It should also be noted that an 800 V jump causes a major change in the curvature of a bimorph and the results above actually correspond to a worst-case scenario. The stabilization times will be even shorter for more realistic (*i.e.* less dramatic) changes to the X-ray beam size.

## 4. Conclusions   

We have provided the first demonstration that piezoelectric bimorph deformable X-ray mirrors can be operated in open loop as high-speed adaptive optics for synchrotron and XFEL applications without the necessity for continuous real-time feedback. Using the newest bimorph mirror design, improved opto-mechanical mounts and a programmable HV power supply operating with a voltage slew rate of up to 300 V s^−1^, we have been able to operate KB mirrors in parallel on a synchrotron beamline to make major changes to the 2D profile of a micro-focused X-ray beam in much less than 1 min.

Both X-ray and visible-light metrology experiments confirm that these timescales can be significantly reduced by minimizing piezoelectric creep. This is achieved by applying small pre-calculated corrective voltages immediately after the main voltage ramp. Corrective voltages are typically only ∼1% of the initial voltage change and allow the required size of the X-ray beam to be reached within ≪10 s. This brings the 2D dynamic performance of X-ray bimorph KB mirrors close to the 1 Hz regime and comparable with the data-collection period of many MX beamlines. Just as importantly, we have shown that the X-ray beam size remains stable indefinitely and that the focus/defocus process is highly repeatable.

The magnitude of the creep-correction voltages is shown to be linearly dependent on the voltage jump, thereby providing a simple method of automatically changing and stabilizing the X-ray beam size and shape without the need for continuous monitoring and feedback. Ideally, this procedure is hidden from the scientific user, who simply requests the desired size of X-ray beam. The ability to change the X-ray beam size rapidly and continuously, whilst preserving the flux delivered to the sample, could be of great benefit to many scientific users at synchrotron and XFEL facilities.

In the near future, we plan to enhance the control software further to enable the beamline user to apply unique sets of voltages rapidly to each piezo electrode, create smoother defocused X-ray beams and automatically compensate creep for each piezo. We also plan to accelerate this process, whereby voltages can be applied to the bimorph’s electrodes at tens of hertz, thereby rapidly changing the size and shape of the X-ray beam. This could have many useful applications, including matching the X-ray beam to the cross-sectional size of an irregularly shaped sample as it is rotated during data collection, rapidly probing variable-sized sub-volumes of larger samples, or catering for different-sized samples as they are rastered through the X-ray beam in quick succession. If changes in the X-ray beam size can be achieved on timescales comparable with the duration of data collection and sample exchange, this means that the ‘dead time’ associated with optimizing the X-ray beam can be considered negligible.
